# Targeting the EZH2-PPAR Axis Is a Potential Therapeutic Pathway for Pancreatic Cancer

**DOI:** 10.1155/2021/5589342

**Published:** 2021-07-22

**Authors:** Jilong Hu, Zhinan Zheng, Jia Lei, Yuxin Cao, Qiyun Li, Zhi Zheng, Chuanjun Chen

**Affiliations:** ^1^Department of Abdominal Surgery Oncology, Jiangxi Cancer Hospital of Nanchang University, Nanchang, Jiangxi 330029, China; ^2^Department of Pharmacy, Jiangxi Cancer Hospital of Nanchang University, Nanchang, Jiangxi 330029, China; ^3^Haiyuan College, Kunming Medical University, Kunming, Yunnan 650106, China; ^4^Department of Medicine, Nanchang University, Nanchang, Jiangxi 330000, China; ^5^Department of Internal Medicine 5th Division, Jiangxi Cancer Hospital of Nanchang University, Nanchang, Jiangxi 330029, China; ^6^Nanchang Royo Biotech Co., Ltd., Nanchang, Jiangxi 330029, China

## Abstract

Enhancer of zeste homolog 2 (EZH2) is abnormally highly expressed in pancreatic cancer (PC). However, it is not ideal to treat PC by inhibiting EZH2. This study reported that the combined use of pan-peroxisome proliferator-activated receptor (PPAR) agonist could significantly improve the anti-PC effect of EZH2 inhibitor. In vitro, PC cell lines PANC-1 and AsPC-1 were cultured, and MTT and flow cytometry were performed to observe the effects of pan-PPAR agonist bezafibrate and EZH2 selective inhibitor GSK126 on cell viability and apoptosis. In vivo, CDXs of PANC-1 and AsPC-1 were established to observe the effects of bezafibrate and GSK126 on bearing tumors. Western blotting was performed to detect the protein expressions of H3K27me3, *β*-catenin, p-*β*-catenin, cyclin D1, c-Myc, and cleaved caspase 3 in vitro and in vivo. The results showed that bezafibrate significantly improved the effects of GSK126 on proliferation inhibition and apoptosis promotion in vitro and the growth suppression of CDX tumors in vivo. It also significantly enhanced the effects of GSK126 on upregulating the expression level of p-*β*-catenin and that of cleaved caspase 3 in vitro and in vivo. In parallel, downregulation of the expression levels of H3K27me3, *β*-catenin, cyclin D1, and c-Myc was also observed in vitro or in vivo. These results suggest that the combination of bezafibrate and GSK126 has synergistic effects on PC, and the molecular mechanism may be related to the enhanced inhibition of the Wnt/*β*-catenin signaling pathway. We believe that targeting the EZH2-PPAR axis is a potential therapeutic pathway for PC.

## 1. Introduction

The incidence of pancreatic cancer (PC) ranks 14th in the world, and it has become the 7th leading cause of cancer death in the world due to its extremely high degree of malignancy [1]. The incidence of PC in China has been increasing year by year, and it ranked the 6th cause of cancer death in the country in 2015 [2]. PC has a short course of disease and rapid development. Its early characteristics are always not obvious, and it is already at an advanced stage at the time of diagnosis. In addition, it is difficult to treat in the late stage, which makes high clinical mortality rate of PC [3]. At present, surgical resection is the only effective method for patients with PC to obtain a cure and long-term survival, while most patients with PC lose the opportunity for surgery due to the late disease stage [4]. What is more, the molecular mechanism of PC is still unclear, which limits the drug development and precise treatment of PC [4, 5].

Gene expression patterns determine the fate of cells, and abnormal gene transcriptions affect the phenotype of cancers [6]. Epigenetic regulation of transcription is a complex dynamic and plastic process, and epigenetic disorder is one of the basic characteristics of cancers [7]. The family of polycomb group genes (PCGs) is a group of important epigenetic regulators that suppress transcription. Polycomb repressive complex 2 (PRC2) is one of the core protein complexes of PCGs including enhancer of zeste 2 (EZH2), suppressor of zeste 12 (Suz12), embryonic ectoderm development (Eed), and the histone binding proteins RbAp46/RbAp48 and mediates gene silencing by regulating chromatin structure [8]. EZH2 is an enzyme catalytic subunit of PRC2 and mediates methylation of Lys27 in histone 3 (H3K27me), including mono-H3K27me (H3K27me1), di-H3K27me (H3K27me2), and tri-H3K27me (H3K27me3) [9, 10]. This methylated H3K27me3 chromatin mark is commonly associated with silencing of differentiation genes in organisms ranging from plants to flies to humans [11]. More and more evidences show that EZH2 is involved in the abnormal transcription of cancer cells and is related to the poor prognosis of a variety of human malignancies. Overexpression of EZH2 leads to increases in H3K27me3, which inhibits the genes related to tumor suppression and cell differentiation (among numerous others) [12]. Inhibiting EZH2 has potential therapeutic effects on a variety of cancers [13]. Huang et al. have confirmed the abnormal overexpression of EZH2 in PC and found that the EZH2 inhibitors have certain therapeutic effects on PC, while the effects were limited [14]. In the preliminary experiments, we found that the anti-PC effect of EZH2 inhibitor GSK126 can be significantly improved by combining bezafibrate, an agonist of pan-peroxisome proliferator-activated receptors (PPAR). This study reported the therapeutic effect of GSK126 combined with bezafibrate on PC in vitro and in vivo.

## 2. Materials and Methods

### 2.1. Reagents

Bezafibrate (dissolved in DMSO as 50 mg/ml) and GSK126 (dissolved in DMSO as 10 mg/ml) were obtained commercially from Selleckchem (Houston, TX, USA). Antibodies of EZH2, histone H3, histone H3 acetyl K27 (H3K27ac), histone H3 trimethyl Lys27 (H3K27me3), *β*-catenin, phospho-*β*-catenin (p-*β*-catenin), cyclin D1, c-Myc, cleaved caspase 3, and GAPDH were purchased from Abcam (Cambridge, UK). Annexin V-FITC apoptosis detection kit was obtained from eBioscience (San Diego, CA, USA).

### 2.2. Cell Culture

Human normal pancreatic ductal epithelial cell line of H6C7 and human PC cell lines of PANC-1 and AsPC-1 were all purchased from Procell (Wuhan, HB, CHN). The cells of PANC-1 were grown in DMEM with 10% calf bovine serum and 1% penicillin-streptomycin at 37°C with 5% CO_2_ (*v*/*v*). The cells of H6C7 and AsPC-1 were grown in RPMI-1640 with 10% calf bovine serum and 1% penicillin-streptomycin at 37°C with 5% CO_2_ (*v*/*v*). Medium was replaced two to three days, and the cells were passaged when the cell adherence area reached 80% of the culture dish.

### 2.3. Experimental Groups and Treatments In Vitro

Cell lines of PANC-1 and AsPC-1 were carried out as independent experiments and grouped as follows: (1) control group (control), in which the cells were treated with vehicle in medium for 120 h; (2) bezafibrate treatment group (Bez), in which the cells were treated with different concentrations of bezafibrate (0, 0.125, 0.25, 0.5, 1, and 2 mM) in medium for 120 h; (3) GSK126 group (GSK126), in which the cells were treated with different concentrations of GSK126 (0, 2.5, 5, 10, 20, and 40 *μ*M) in medium for 120 h; and (4) bezafibrate combination with GSK126 group (Bez+GSK126), in which the cells were treated with bezafibrate and GSK126 together in medium for 120 h.

### 2.4. MTT Assay

3-(4,5-Dimethylthiazol-2-yl)-5-(3-carboxymethoxyphenyl)-2-(4-sulfophenyl)-2H-tetrazolium (MTT; Promega, WI, USA) was used to detect the cell viability. Briefly, after 120 h treatment in 96-well plates, the cells were incubated with 20 *μ*l MTT (5 mg/ml) in 100 *μ*l cell culture medium for 4 h at 37°C. After 4 h, the absorbance of each well was measured at a wavelength of 490 nm [15].

### 2.5. Apoptosis Assay

Trypsin-EDTA was used to obtain single-cell suspension after incubating for 10 min, and then, the cells were washed with chilled D-Hanks (pH = 7.2 ~ 7.4) after centrifugation and incubated in Annexin-V binding buffer for 15 min at room temperature, containing Annexin-V-FITC. Flow cytometry (Becton Dickinson, USA) was used to quantify the fluorescence of Annexin-V-FITC with a minimum of 10,000 cells counted for each group [15].

### 2.6. Animals

18 to 22 g (4-6 weeks) adult male Balb/c nude mice were obtained from Nanchang University Laboratory Animal Center. Mice were allowed to access food and water *ad libitum* under a 12 h dark/light cycle at 22°C to 25°C. Experiments were carried out according to the *Guide for the Care and Use of Laboratory Animals* published by the U.S. National Institutes of Health (NIH Publication No. 85-23, revised in 1996) and approved by the Ethics Committee of Nanchang University (No. 2019-0402).

### 2.7. Cell-Derived Xenograft (CDX)

Lidocaine was applied to the skin of the left forelimb scapula of nude mice after skin preparation by iodophor; then, 100 *μ*l cell suspension (1 × 10^6^/ml) was extracted by a microinjector and injected into the subcutaneous area of the scapula of nude mice (P_0_). The nude mice were fed routinely, and the tumor formation was continuously observed. When the bearing tumor growth reached 1000 mm^3^, CO_2_ euthanasia was performed. In aseptic environment, the tumor was completely peeled off and cut into tissue blocks of uniform size (2 × 2 × 2 mm). After skin preparation with iodophor and local anesthesia with lidocaine, single tissue blocks were inoculated subcutaneously into the subcutaneous area of the scapula of normal nude mice (P_1_). When the bearing tumor growth reached 1000 mm^3^, normal adult mice were inoculated according to the above method (P_2_). CDXs of P_3_ were used for formal experiments.

### 2.8. Experimental Groups and Treatments In Vivo

CDXs of PANC-1 and AsPC-1 were carried out as independent experiments. The P_3_ mouse bearing tumors with tumor volume of about 100-200 mm^3^ were selected and divided into the following groups: (1) control group (control), in which the mice were treated with vehicle daily; (2) bezafibrate treatment group (Bez), in which the mice were treated with bezafibrate (150 mg/kg/d) intraperitoneally; (3) GSK126 group (GSK126), in which the mice were treated with GSK126 (150 mg/kg/d) intraperitoneally; and (4) bezafibrate combination with GSK126 group (Bez+GSK126), in which the mice were treated with bezafibrate and GSK126 as above. The doses of bezafibrate and GSK126 were determined in the preliminary experiments. The tumor volumes and body weights of mice were measured every three days. Animals were euthanized by CO_2_ when the following conditions were met. (1) The weight of mouse was reduced more than 20%. (2) The bearing tumor has obvious ulcer. (3) The volume of tumor was more than 2000 mm^3^. All mice were euthanized humanely on the 21st day.

### 2.9. Western Blotting

Total proteins in cells or bearing tumors of CDXs were extracted by RIPA buffer (Solarbio, Beijing, CHN), electrophoresed on 10% SDS-PAGE gel, and transferred to PVDF membranes. Then, the membranes were blocked with 10% nonfat milk for 60 min and incubated with primary detection antibodies overnight at 4°C. After being washed by TBST, the membranes were incubated with HRP-conjugated secondary antibodies. Immunoreactive bands were detected by enhanced chemiluminescence (ECL, Thermo Fisher Scientific, Waltham, MA, USA) and quantified with the ImageJ v2.1.4.7 software (National Institutes of Health, Bethesda, MD, USA).

### 2.10. Quantitative Polymerase Chain Reaction (qPCR) Assay

Total RNA was extracted from cells using TRIzol reagent (Thermo Fisher, Waltham, MA, USA). Primer 5.0 software was used to design the oligonucleotide primer sequences of EZH2 (F: GGACTCAGAAGGCAGTGGAG, R: CTTGAGCTGTCTCAGTCGCA). GAPDH was used as an internal control. The synthesized first-strand cDNA samples were subjected to qPCR using a SYBR Green PCR Master Mix (Toyobo Bio-Technology, Shanghai, CHN), and the qPCR reaction was performed on an ABI Prism 7700 Sequence Detector (Thermo Fisher). Threshold cycle (Cq) values were determined, and relative fold changes in mRNA expression were calculated using the formula 2^-*ΔΔ*Cq^.

### 2.11. Statistical Analysis

Data was presented as means ± S.E.M. and analyzed by SPSS version 20.0 (IBM Corp., Armonk, NY, USA) for variance homogeneity test and one-way analysis of variance. *P* < 0.05 was considered to indicate a statistically significant difference. Combination index (CI) of drug combination was calculated by CalcuSyn software (Biosoft, Ferguson, MO and Cambridge, UK) using the Chou-Talalay method [16]. Drug combination effects were assessed according to CI, which quantitatively established additivity (CI = 0.9–1.1), synergy (CI < 0.9), and antagonism (CI > 1.1). The resulting values were utilized in the construction of a plot of CI values over a range of affected fractions (Fa-CI plot) [17].

## 3. Results

### 3.1. EZH2 Is Highly Expressed in Cells of PANC-1 and AsPC-1

**Figure 1:**
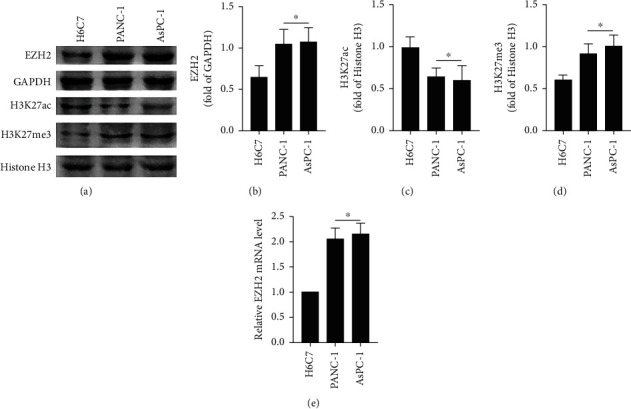
The protein expression levels of EZH2, H3K27ac, and H3K27me3 in cells of PANC-1 and AsPC-1. (a) Representative bands of western blot of EZH2, H3K27ac, H3K27me3, and histone H3 in cells of H6C7, PANC-1, and AsPC-1. (b) The relative band intensity of EZH2 in cells of H6C7, PANC-1, and AsPC-1. (c) The relative band intensity of H3K27ac in cells of H6C7, PANC-1, and AsPC-1. (d) The relative band intensity of H3K27me3 in cells of H6C7, PANC-1, and AsPC-1. (e) The relative mRNA level of EZH2 in cells of H6C7, PANC-1, and AsPC-1. The values were expressed as the means ± S.E.M. (*n* = 6 for each group). ^∗^*P* < 0.05 vs. the H6C7 group.

Western blotting was used to detect the protein expressions of EZH2, H3K27ac, and H3K27me3 in cells of H6C7, PANC-1, and AsPC-1. As shown in Figures 1(a)–1(d), compared with H6C7, the proteins of EZH2 and H3K27me3 were significantly upregulated in PANC-1 and AsPC-1, while H3K27ac was significantly downregulated (*P* < 0.05). The qPCR was used to detect the mRNA transcription level of EZH2, and the result showed that the relative mRNA levels of EZH2 were significantly upregulated in PANC-1 and AsPC-1 (Figure 1(e)), which is consistent with the results of western blotting.

### 3.2. Effects of Combination of Bezafibrate and GSK126 on Cell Viability of PANC-1 and AsPC-1

**Figure 2:**
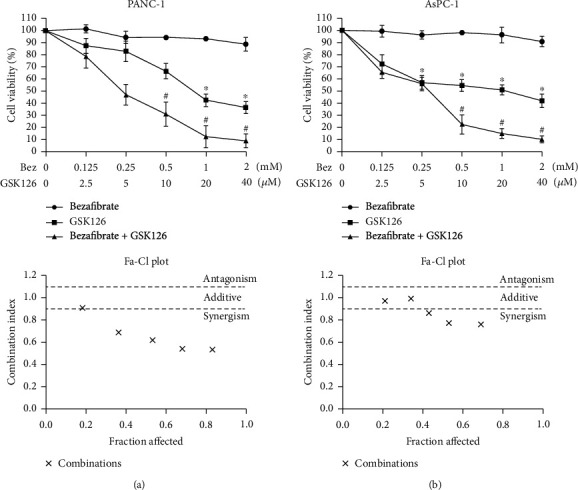
Effects of combination of bezafibrate and GSK126 on cell viability of PANC-1 and AsPC-1. (a) Effects of different concentrations of bezafibrate, GSK126, and their combination on cell viability of PANC-1 after 120 h treatment. Combination indexes were calculated accordingly on the indicated concentrations of bezafibrate and GSK126. (b) Effects of different concentrations of bezafibrate, GSK126, and their combination on cell viability of PANC-1 after 120 h treatment. Combination indexes were calculated accordingly on the indicated concentrations of bezafibrate and GSK126. The values were expressed as the means ± S.E.M. (*n* = 6 for each group). ^∗^*P* < 0.05 vs. the bezafibrate group; ^#^*P* < 0.05 vs. the GSK126 group.

Different concentrations of bezafibrate or GSK126 were used to treat PANC-1 and AsPC-1 cells. As shown in Figure 2, when the concentration of bezafibrate reached 2 mM, the cell viability was significantly inhibited. GSK126 could significantly inhibit the viability of cells in a dose-dependent manner, and the IC50 of PANC-1 and AsPC-1 was 16 *μ*M and 24 *μ*M, respectively. What is important, the combination of bezafibrate and GSK126 could inhibit cell viability in a dose-dependent manner. Their corresponding CI plot analysis showed that the combination of bezafibrate and GSK126 inhibited cell viability synergistically at the majority of concentrations. The combined drug treatment of 1 mM bezafibrate and 20 *μ*M GSK126 marked a transition from drug concentrations that prevented the growth of cancer cells only to a concentration that effectively prevented the growth of cancer cells. Therefore, these concentrations were examined further.

### 3.3. Effects of Combination of Bezafibrate and GSK126 on Apoptosis in Cells of PANC-1 and AsPC-1

**Figure 3:**
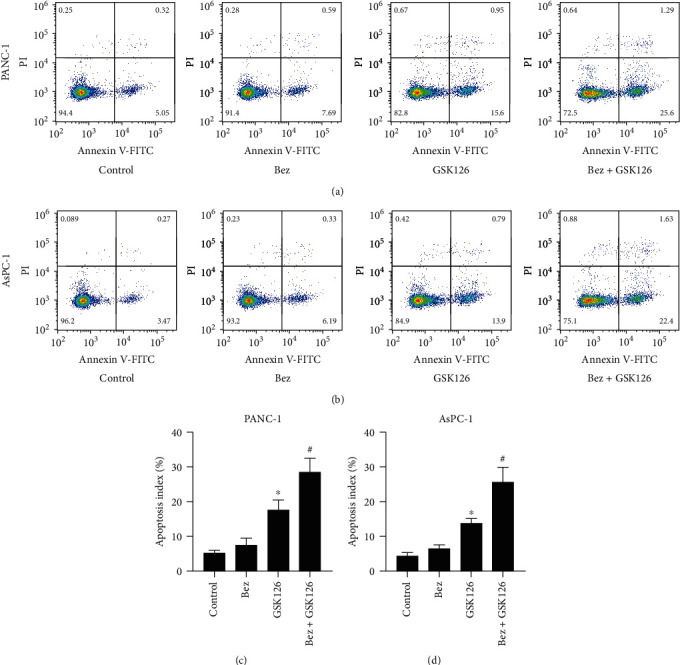
Effects of combination of bezafibrate and GSK126 on apoptosis of PANC-1 and AsPC-1. The concentrations of bezafibrate and GSK126 were 1 mM and 20 *μ*M, respectively. (a) The representative images of flow cytometry in each experimental group of PANC-1 cells. (b) The representative images of flow cytometry in each experimental group of AsPC-1 cells. (c) Apoptosis rates in each experimental group of PANC-1 cells. (d) Apoptosis rates in each experimental group of AsPC-1 cells. The values were expressed as the means ± S.E.M. (*n* = 6 for each group). ^∗^*P* < 0.05 vs. control; ^#^*P* < 0.05 vs. the GSK126 group.

As shown in Figure 3, bezafibrate alone could not significantly promote the apoptosis of PANC-1 or AsPC-1 compared with the control (*P* > 0.05), while GSK126 alone treatment could significantly increase the apoptosis levels of PANC-1 and AsPC-1 (*P* < 0.05 vs. the control group). The combination of bezafibrate and GSK126 could enhance the proapoptotic effects of GSK126 on PANC-1 or AsPC-1 significantly (*P* < 0.05 vs. the GSK126 group).

### 3.4. Effects of Combination of Bezafibrate and GSK126 on Protein Expressions in Cells of PANC-1 and AsPC-1

**Figure 4:**
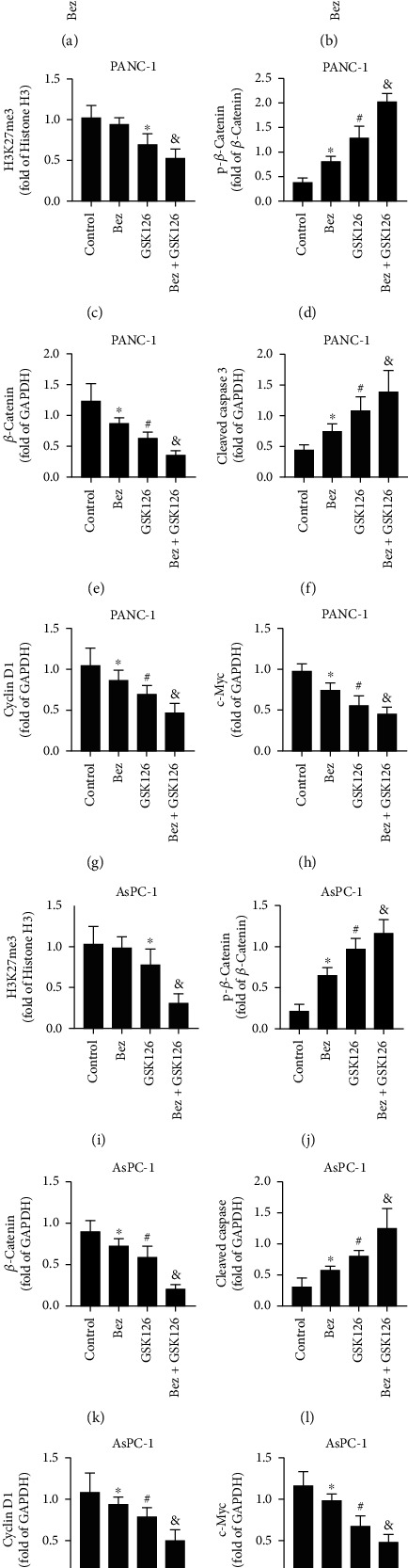
Effects of combination of bezafibrate and GSK126 on protein expressions in cells of PANC-1 and AsPC-1. (a) Representative bands of western blot of H3K27me3, p-*β*-catenin, *β*-catenin, and cleaved caspase 3 in cells of PANC-1. (b) Representative bands of western blot of H3K27me3, p-*β*-catenin, *β*-catenin, and cleaved caspase 3 in cells of AsPC-1. (c) The relative band intensity of H3K27me3 in each experimental group of PANC-1 cells. (d) The relative band intensity of p-*β*-catenin in each experimental group of PANC-1 cells. (e) The relative band intensity of *β*-catenin in each experimental group of PANC-1 cells. (f) The relative band intensity of cleaved caspase 3 in each experimental group of PANC-1 cells. (g) The relative band intensity of cyclin D1 in each experimental group of PANC-1 cells. (h) The relative band intensity of c-Myc in each experimental group of PANC-1 cells. (i) The relative band intensity of H3K27me3 in each experimental group of AsPC-1 cells. (j) The relative band intensity of p-*β*-catenin in each experimental group of AsPC-1 cells. (k) The relative band intensity of *β*-catenin in each experimental group of AsPC-1 cells. (l) The relative band intensity of cleaved caspase 3 in each experimental group of AsPC-1 cells. (m) The relative band intensity of cyclin D1 in each experimental group of AsPC-1 cells. (n) The relative band intensity of c-Myc in each experimental group of AsPC-1 cells. The values were expressed as the means ± S.E.M. (*n* = 6 for each group). ^∗^*P* < 0.05 vs. the control group, ^#^*P* < 0.05 vs. the Bez group, and ^&^*P* < 0.05 vs. the GSK126 group.

As shown in Figure 4, bezafibrate alone has no significant effect on H3K27me3 (*P* > 0.05 vs. the control group), while GSK126 could significantly downregulate the expression level of H3K27me3 (*P* < 0.05 vs. the control group). Bezafibrate could enhance the inhibitory effect of GSK126 on EZH2 methyltransferase and significantly further downregulate the expression of H3K27me3 (*P* < 0.05 vs. the GSK126 group). Both bezafibrate and GSK126 could significantly upregulate the expressions of p-*β*-catenin and cleaved caspase 3 and downregulate the expressions of *β*-catenin, cyclin D1, and c-Myc (*P* < 0.05 vs. the control group), and the combination of bezafibrate and GSK126 could further affect the expressions of above proteins accordingly (*P* < 0.05 vs. the GSK126 group).

### 3.5. Effects of Combination of Bezafibrate and GSK126 on CDX Tumor Volumes

**Figure 5:**
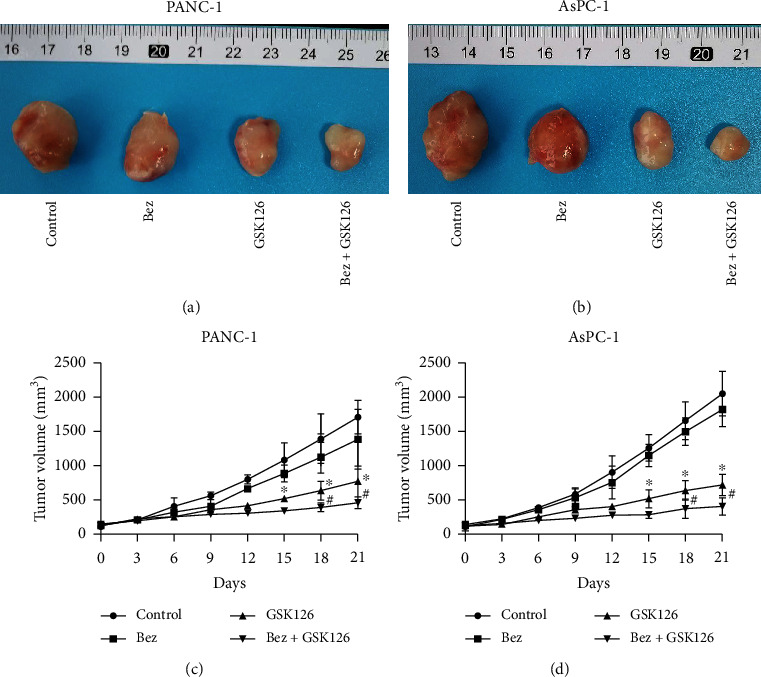
Effects of combination of bezafibrate and GSK126 on CDX tumor volumes. (a) Representative image of bearing tumors of PANC-1 in each experimental group. (b) Representative image of bearing tumors of AsPC-1 in each experimental group. (c) Growth curves of bearing tumors of PANC-1 in each experimental group. (d) Growth curves of bearing tumors of AsPC-1 in each experimental group. The values were expressed as the means ± S.E.M. (*n* = 6 for each group). ^∗^*P* < 0.05 vs. control; ^#^*P* < 0.05 vs. the GSK126 group.

The effects of bezafibrate and GSK126 on mice bearing PANC-1 and AsPC-1 tumors are shown in Figure 5. Bezafibrate treatment alone has no obvious effects on inhibiting the growth of bearing tumors of PANC-1 or AsPC-1 (*P* > 0.05 vs. the control group), while GSK126 treatment alone could significantly inhibit the growth of bearing tumors of PANC-1 or AsPC-1 (*P* < 0.05 vs. the control group). When comparing with the group of GSK126, bezafibrate in combination with GSK126 could more significantly inhibit the growth of bearing tumors of PANC-1 or AsPC-1 (*P* < 0.05).

### 3.6. Effects of Combination of Bezafibrate and GSK126 on Protein Expressions in CDXs

**Figure 6:**
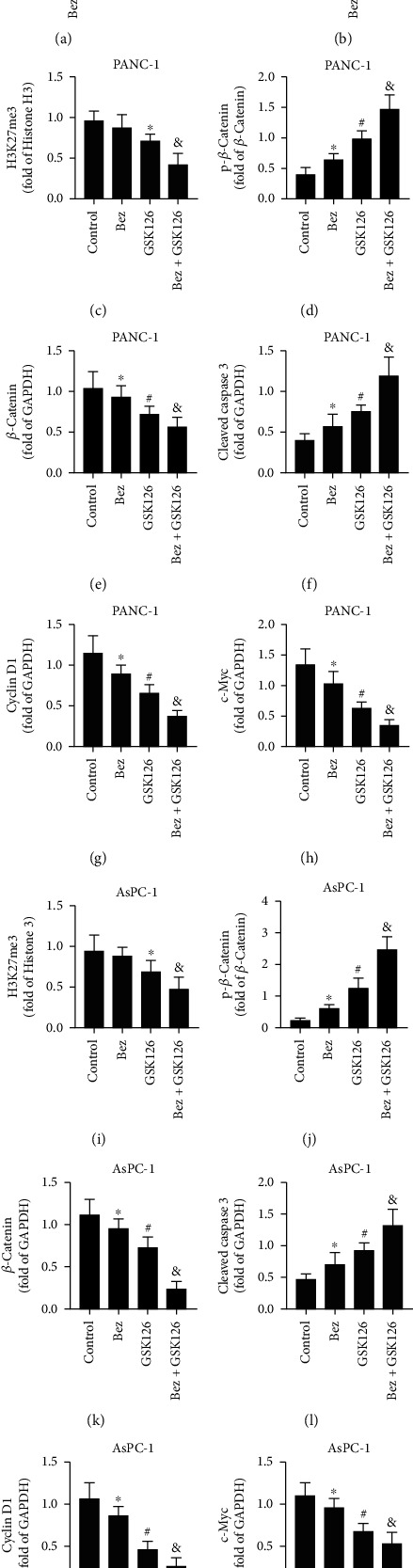
Effects of combination of bezafibrate and GSK126 on protein expressions in bearing tumors of CDXs. (a) Representative bands of western blot of H3K27me3, p-*β*-catenin, *β*-catenin, and cleaved caspase 3 in bearing tumors of PANC-1 CDX. (b) Representative bands of western blot of H3K27me3, p-*β*-catenin, *β*-catenin, and cleaved caspase 3 in bearing tumors of AsPC-1 CDX. (c) The relative band intensity of H3K27me3 in each experimental group of PANC-1 CDX. (d) The relative band intensity of p-*β*-catenin in each experimental group of PANC-1 CDX. (e) The relative band intensity of *β*-catenin in each experimental group of PANC-1 CDX. (f) The relative band intensity of cleaved caspase 3 in each experimental group of PANC-1 CDX. (g) The relative band intensity of cyclin D1 in each experimental group of PANC-1 CDX. (h) The relative band intensity of c-Myc in each experimental group of PANC-1 CDX. (i) The relative band intensity of H3K27me3 in each experimental group of AsPC-1 CDX. (j) The relative band intensity of p-*β*-catenin in each experimental group of AsPC-1 CDX. (k) The relative band intensity of *β*-catenin in each experimental group of AsPC-1 CDX. (l) The relative band intensity of cleaved caspase 3 in each experimental group of AsPC-1 CDX. (m) The relative band intensity of cyclin D1 in each experimental group of AsPC-1 CDX. (n) The relative band intensity of c-Myc in each experimental group of AsPC-1 CDX. The values were expressed as the means ± S.E.M. (*n* = 6 for each group). ^∗^*P* < 0.05 vs. the control group, ^#^*P* < 0.05 vs. the Bez group, and ^&^*P* < 0.05 vs. the GSK126 group.

As shown in Figure 6, bezafibrate treatment has no obvious effects on methylation of histone H3, and GSK126 treatment could significantly prevent the methylation of histone H3 and downregulate the expression level of H3K27me3 (*P* < 0.05 vs. the control group). The combination of bezafibrate and GSK126 could further downregulate the expression level of H3K27me3 (*P* < 0.05 vs. the GSK126 group). Both bezafibrate and GSK126 could significantly upregulate the expressions of p-*β*-catenin and cleaved caspase 3 and downregulate the expressions of *β*-catenin, cyclin D1, and c-Myc (*P* < 0.05 vs. the control group), and the combination of bezafibrate and GSK126 could further affect the expressions of above proteins accordingly (*P* < 0.05 vs. the GSK126 group).

## 4. Discussion

In this study, we found that EZH2 was abnormally overexpressed in PC cell lines, suggesting that EZH2 is closely related to PC. GSK126, a selective inhibitor of EZH2, can significantly inhibit the proliferation and promote the apoptosis of PC cells. The pan-PPAR agonist bezafibrate alone has no significant effect on the proliferation and apoptosis of PC, but its combined use with GSK126 could further enhance the anti-PC cancer effect, suggesting that GSK126 and bezafibrate have synergistic effects at certain concentrations. To our knowledge, this is the first report on the synergistic effect of GSK126 and bezafibrate on PC.

Peroxisomes were first discovered in the liver of rats and participated in cellular functions such as fatty acid metabolic transport and ROS detoxification [18]. Peroxisome proliferators can induce the proliferation of peroxisomes, and the receptors of proliferators are generally referred to as PPARs, which are involved in activating/inhibiting target genes [19]. PPARs contain three subtypes including *γ*, *α*, and *β*/*δ*, which are widely expressed in human cells [20]. PPARs play important roles in the regulation of cell differentiation, development, metabolism, and tumorigenesis. Pro12Ala polymorphism in PPAR *γ* was found to be a risk factor for gastric cancer [21]. PPAR *α* and *γ* have been found to be associated with the risk of breast cancer [22]. Recent studies have shown that the expression levels of PPAR pathway genes MED1, PRKCA, and PRKCB in PC are higher than normal tissues, suggesting that PPAR may contribute to susceptibility to PC [23]. In mammalian cells, PPARs and the Wnt/*β*-catenin signaling pathway have the opposite manner. PPAR agonists can inhibit nuclear translocation of *β*-catenin, thus limiting the regulation of the Wnt/*β*-catenin signaling pathway, and conversely, the inhibition of the Wnt/*β*-catenin signaling pathway can induce PPAR activation [24, 25]. Wnt/*β*-catenin signal plays an important role in cell fate, epithelial-mesenchymal transition signal transduction, and embryonic development. Its dysfunction involves a variety of diseases, including PC [26]. PPAR agonists are commonly used in the treatment of diabetes. Since the target organ of both diabetes and PC is the pancreas, studies have reported the therapeutic effects of PPAR agonists on PC. However, simply activating PPARs cannot achieve exciting results [27, 28]. In this study, we observed that bezafibrate alone, an agonist of pan-PPAR, had certain inhibitory effects on PC cells PANC-1 and AsPC-1 in vivo and in vitro with no significance. Western blotting results showed that bezafibrate could significantly inhibit the protein expressions of *β*-catenin, cyclin D1, and c-Myc and increase the expression of p-*β*-catenin in PANC-1 and AsPC-1. These suggested that activation of PPARs has a limited therapeutic effect on PC, and its limited effect may be related to its ability to inhibit the activity of the Wnt/*β*-catenin pathway in PC cells.

Studies have shown that the EZH2-PRC2 complex controls multiple target genes in the Wnt pathway at the epigenetic level [29]. In cervical cancer, EZH2 activates the Wnt/*β*-catenin pathway through epigenetic silencing of GSK-3*β* and TP53, thereby promoting cervical cancer cell proliferation and tumor formation [30]. In the mechanism of EZH2-mediated activation of Wnt/*β*-catenin signal to promote carcinogenesis, EZH2 acts as a transcription repressor, inhibiting Wnt antagonists by reducing the acetylation of H3K27, thereby promoting the development of different types of cancers [31, 32]. Conversely, EZH2 knockout can induce G2/M phase arrest of breast cancer and regulate the expressions of cyclin D1 and *β*-catenin [33]. By inhibiting EZH2 to interfere with the Wnt/*β*-catenin pathway, the progression of glioma can be inhibited [34]. Inhibition of EZH2 can also reduce the expression of apoptosis-inhibiting genes by inhibiting mTOR activity and aggravate cisplatin-induced apoptosis [35]. In this study, the EZH2 inhibitor GSK126 can significantly inhibit the methylation level of histone H3, prevent the proliferation of PC cells in vitro and in vivo, and promote the expressions of apoptosis-related protein to aggravate apoptosis of cancer cells. Meanwhile, GSK126 could significantly downregulate the expression of Wnt/*β*-catenin pathway key protein *β*-catenin and promote its phosphorylation. It should be noted that *β*-catenin is bound to and phosphorylated by a destructive complex that resides within the cytoplasm. Upon phosphorylation, *β*-catenin leaves the complex to be further ubiquitinated by beta-transducin repeat-containing proteins and degraded by the proteasome [36]. Combining with bezafibrate can significantly enhance the anti-PC effects of GSK126 and further inhibit the Wnt/*β*-catenin signaling pathway. We speculate that the synergistic anti-PC effects of GSK126 and bezafibrate are related to their ability to synergistically inhibit the activity of the Wnt/*β*-catenin pathway.

In conclusion, we have proved that bezafibrate, an agonist of pan-PPAR, can significantly enhance the proliferation inhibition and apoptosis promotion of GSK126, a selective inhibitor of EZH2, on PC cells in vivo and in vitro. The combination of bezafibrate and GSK126 enhanced the inhibition of Wnt/*β*-catenin pathway activity, which may be the molecular mechanism of bezafibrate and GSK126 in synergistic anti-PC. The above results suggest that targeting the EZH2-PPAR axis may be a potential treatment for PC. However, the biggest limitation of this study is inadequate exploration in the possible protein interaction between EZH2 and PPAR, which will be the focus of future research.

## Data Availability

The datasets generated and analyzed during the study are available from the corresponding author on request.
